# The Impact of Milk and Its Components on Epigenetic Programming of Immune Function in Early Life and Beyond: Implications for Allergy and Asthma

**DOI:** 10.3389/fimmu.2020.02141

**Published:** 2020-10-21

**Authors:** Betty C. A. M. van Esch, Mojtaba Porbahaie, Suzanne Abbring, Johan Garssen, Daniel P. Potaczek, Huub F. J. Savelkoul, R. J. Joost van Neerven

**Affiliations:** ^1^Division of Pharmacology, Utrecht Institute for Pharmaceutical Sciences, Utrecht University, Utrecht, Netherlands; ^2^Danone Nutricia Research, Utrecht, Netherlands; ^3^Cell Biology and Immunology Group, Wageningen University & Research, Wageningen, Netherlands; ^4^Institute of Laboratory Medicine, Member of the German Center for Lung Research (DZL), The Universities of Giessen and Marburg Lung Center (UGMLC), Philipps-University Marburg, Marburg, Germany; ^5^John Paul II Hospital, Krakow, Poland; ^6^FrieslandCampina, Amersfoort, Netherlands

**Keywords:** epigenetics, epigenetic imprinting, environmental factors, unprocessed (raw) milk, breastfeeding, allergy, nutritional programming, bioactive milk components

## Abstract

Specific and adequate nutrition during pregnancy and early life is an important factor in avoiding non-communicable diseases such as obesity, type 2 diabetes, cardiovascular disease, cancers, and chronic allergic diseases. Although epidemiologic and experimental studies have shown that nutrition is important at all stages of life, it is especially important in prenatal and the first few years of life. During the last decade, there has been a growing interest in the potential role of epigenetic mechanisms in the increasing health problems associated with allergic disease. Epigenetics involves several mechanisms including DNA methylation, histone modifications, and microRNAs which can modify the expression of genes. In this study, we focus on the effects of maternal nutrition during pregnancy, the effects of the bioactive components in human and bovine milk, and the environmental factors that can affect early life (i.e., farming, milk processing, and bacterial exposure), and which contribute to the epigenetic mechanisms underlying the persistent programming of immune functions and allergic diseases. This knowledge will help to improve approaches to nutrition in early life and help prevent allergies in the future.

## Introduction

**Figure 1:**
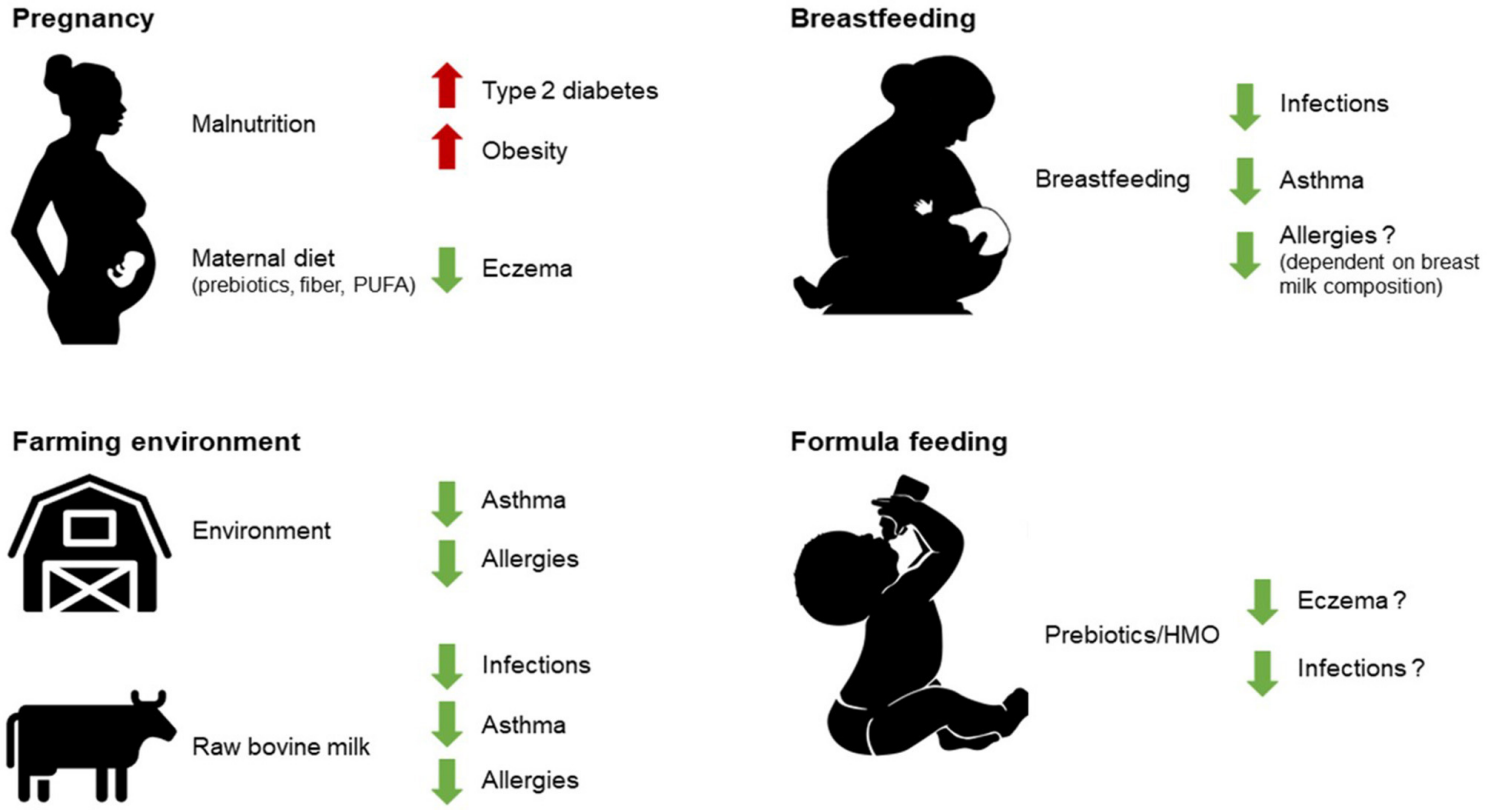
As described in this review, early life nutrition (breastfeeding, raw milk consumption, and some infant formula components), early life environmental exposures (such as farming environment), as well as prenatal development under the influence of maternal diet can all have sustained effects on health outcomes later in life. PUFA, polyunsaturated fatty acids; HMO, human milk oligosaccharides.

There is increasing evidence to suggest that maternal diet during pregnancy, breastfeeding, early life nutrition, and early life malnutrition can have sustained effects on immunological outcomes, such as respiratory allergies, and metabolic outcomes such as type 2 diabetes and obesity. Nutritional programming during gestation might permanently affect the immunological competence and nutritional status in early life Figure 1. This is exemplified by the thrifty phenotype, where the metabolic response to undernutrition during the fetal period is inappropriate during overnutrition later in life, leading to disease manifestations (1). Several studies have since shown that prenatal exposure to famine is associated with the development of type 2 diabetes later in life (2–4), and an epigenetic link was demonstrated in relation to the Dutch hunger winter where epigenetic modification of the IGF2 gene was shown to be linked to famine during prenatal development (5).

Epigenetic mechanisms may play an important role in these effects. It has even been suggested that early life nutrition forms the basis for susceptibility to a plethora of chronic age-related non-communicable diseases (NCD), like respiratory allergies (6–9). Thus, specific and adequate nutrition during pregnancy and early life are considered important factors that could reduce instances of allergic diseases. Epidemiologic and experimental studies show that nutrition is important for (immunological) health, especially when we are very young and during prenatal development, which may influence health and disease throughout our lives (6, 10). The structures of the mucosal immune system in the gastrointestinal (GI) tract are fully developed *in utero* by gestational week 28 (11). Increasing evidence suggests that maternal diet and other prenatal exposures can influence this development by crossing the placenta (12–14). In the first year of life, the mucosal immune system is further shaped by microbial colonization and oral feeding (15). Breastfeeding is the normal way of providing newborns with nutrients for healthy growth and development and a diet exclusively comprised of breastfeeding has various beneficial outcomes, such as reducing the risk of GI diseases, allergies, colitis, and respiratory infections (16). Besides conferring protection against these short-term outcomes, breastfeeding also reduces the long-term risks of developing diseases like type 2 diabetes and obesity (17). In analogy to breast milk, raw, unprocessed, bovine milk is a rich source of immunomodulatory components (18–20). Studies have indicated that it may protect against common respiratory infections in infants that consume unprocessed bovine milk (21). In addition, epidemiological evidence shows a clear association between the consumption of raw cow's milk and the prevention of allergy development (22–29). Epigenetic mechanisms that are regulated by many immune processes can thereby influence the course of allergic diseases.

Epigenetic mechanisms (Box 1) and transcription regulatory factors allow a flexible adaptation in the fetus. They neonate to a fluctuating external environment whereby heritable, non-DNA encoded, alterations in gene expression patterns occur. Especially relevant in early life, several factors drive the epigenetic changes that occur throughout life: environment (e.g., exposure to microbial components in inhaled dust), diet (e.g., components present in breast milk and bovine milk), and the GI microbiota and its metabolites (e.g., through the production of short-chain fatty acids [SCFA] after fermentation of dietary non-digestible oligosaccharides). Thus, environmental, dietary, and microbiota-derived epigenetic modifications during gestation and early life can shape future immunity to the development of diseases like obesity, type 2 diabetes, allergy, asthma, and infections. Most of our current knowledge on the environmental and dietary effects on epigenetics and early life immune function comes from epidemiological findings which indicate that children growing up on farms have a decreased risk of developing allergies, especially asthma. For this reason, we will focus this review on the effects of maternal nutrition during pregnancy, the effects of bioactive components in human and bovine milk, and the environmental factors in early life that can contribute to the epigenetic mechanisms involved in the course of allergic diseases.

Box 1 **Epigenetic mechanisms.**

**Figure:**
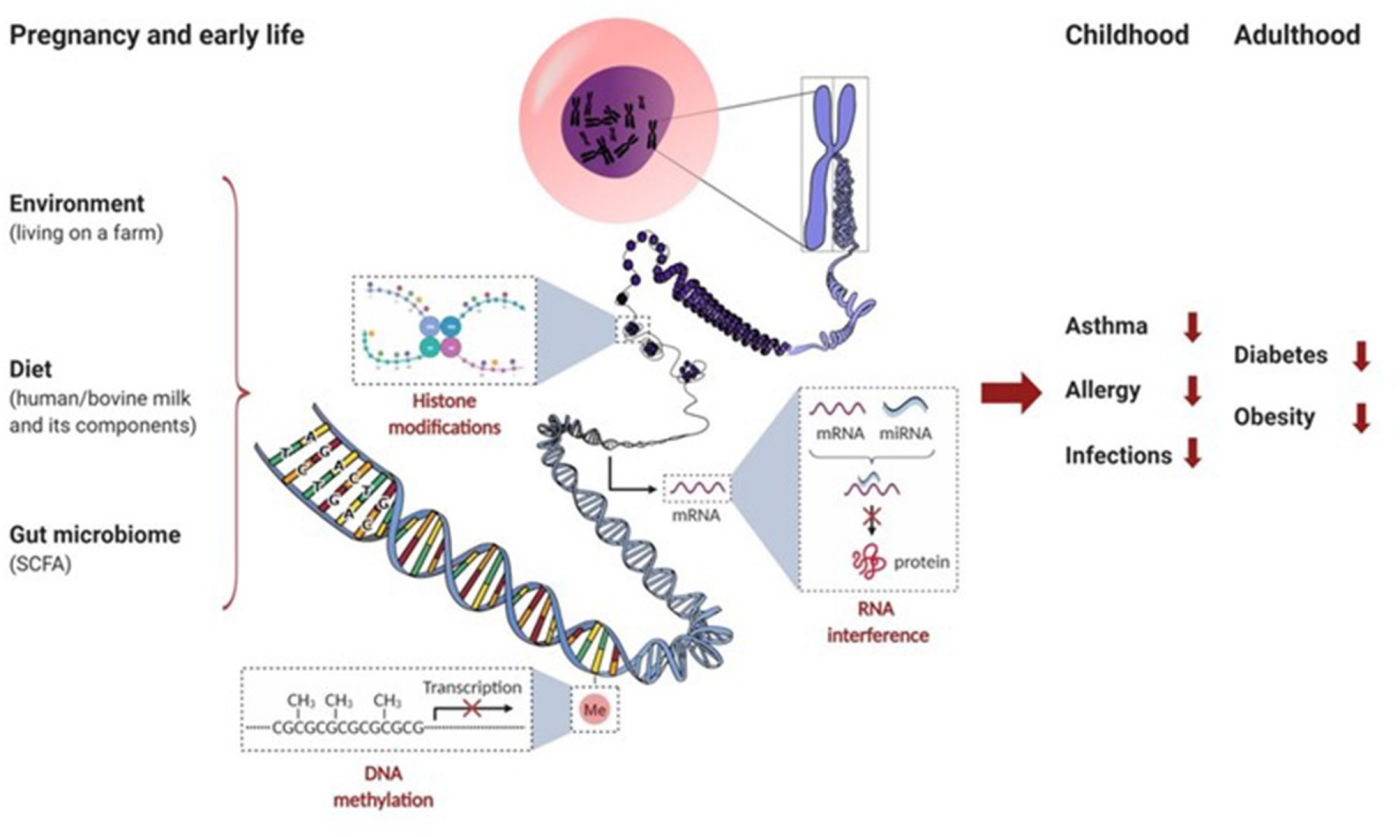

Epigenetics refers to systems that control gene expression in a heritable fashion without changing the genomic sequences. The epigenome is much more flexible than the genome and shows different phenotype variations that are influenced by environmental factors and dietary habits. Epigenetic mechanisms include DNA methylation, histone modifications, and RNA interference by microRNAs (miRNAs) (See in this Box figure). Epigenetic mechanisms thus contribute to the regulation of gene expression at the level of transcription by DNA methylation and by modifying chromatin accessibility through posttranslational modifications of histones, and after transcription by mRNA silencing. These epigenetic mechanisms can regulate gene expression by modifying the accessibility of the DNA to transcription enzymes without altering the DNA nucleotide sequence, influencing stability of mRNA or translation efficiency, and others (30–32). The transfer of a methyl group onto DNA, performed by DNA methyltransferases (DNMTs), can directly regulate the rate of gene transcription. DNA demethylation is catalyzed by several enzymes serving as controllers for the equilibrium of DNA methylation (33). For example, methylation of DNA in the promoter regions of cytokines can influence immune responsiveness by steering Th cell differentiation into Th1, Th2, Th17, or Treg (34, 35). For more details see Box 2. In addition, histone modifications like acetylation, methylation, phosphorylation and others can also modulate the development and activity of immune cells. Histone acetylation is an important remodeling activity that is catalyzed by a series of enzymes called histone acetyltransferases (HATs). Acetylation is generally considered as a permissive activity that facilitates gene transcription. On the contrary, histone deacetylases (HDACs) reverse HAT activity and tighten up the folding of DNA around the histones and make them less accessible for transcription factors (31, 36). The interplay between HATs and HDACs determines the histone acetylation balance and regulates the gene expression (37, 38) and production of pro-inflammatory (IL-1β, IL-5, IL-6, IL-8, IL-12, and TNFα) and anti-inflammatory mediators (IL-10). Histone methyltransferases (HMTs) and demethylases (HDMs) serve as controller enzymes for the equilibrium of histone methylation (31). Finally, RNA interference can occur by small noncoding RNAs, most notably miRNAs that are found in biological fluids as well as in extracellular vesicles (e.g., in milk). MiRNAs represent short noncoding RNA molecules of 18 to 23 nucleotides that control gene expression by inducing mRNA degradation and/or inhibit post-transcriptional translation. As a result, specific miRNA can silence selective gene expression (32). For example, milk contains extracellular vesicles or exosomes that contain a wide range of microRNAs, including miR-21, miR-29b, miR-148a, and miR-155 that is known to influence Foxp3 expression and Treg development (39).

## Epigenetic Regulation of TH2 Development in Allergic Disease

Epigenetic changes have been strongly associated with allergies and asthma and might thereby serve as biomarkers. The role of epigenetic mechanisms, particularly DNA methylation, in allergic diseases is at the interface of gene regulation, environmental stimuli, and developmental processes, thereby determining the pathogenesis of asthma and allergy. Alterations of the DNA methylation status in the genes specific for a different subset of T helper (Th) cells that are considered to be a good example of how epigenetic modulation can influence the development of asthma and other allergic diseases.

The differentiation of naïve CD4+ T cells into Th subpopulations is strictly regulated, with changes in epigenetic marks at main lineage-determining loci encoding transcription factors like GATA3, RORγt, TBX21, and Foxp3 playing a pivotal role. These changes affect the differentiation into mature Th subpopulations, such as Th1, Th2 (and Th9), regulatory T cells (Treg cells), and Th17 (30, 35, 47, 48). In naïve CD4+ T cells, which express a moderate level of GATA3 mRNA after receiving signals via the T cell receptors (TCRs) in the presence of IL-4, activated STAT6 proteins bind to the GATA3 gene locus, driving Th2 differentiation, which is a characteristic in the development of allergy. Differentiation of human CD4+ cells into the Th2 subtype is accompanied by the induction of DNase I hypersensitive (DHS) sites and CpG demethylation around these (DHS) regions within the IL-4 and IL-13 promoters. Extensive studies of the Th2 cytokine locus control region have shown that specific sites undergo rapid demethylation during Th2 differentiation (49).

In addition to DNA methylation, histone modifications are also important in guiding T-cell differentiation. T-bet and GATA3 transcription factors control lineage-specific histone acetylation of IFN-γ and IL-4 loci during Th1/Th2 differentiation. Rapid methylation of H3K9 and H3K27 residues (repressive marks) at the IFN-γ locus was associated with differentiating toward Th1 cells, while demethylation of H3K9 and methylation of H3K27 was associated with Th2 differentiation (49). Epithelial alarmins (IL-25, IL-33, thymic stromal lymphopoietin [TSLP]) induce an inflammatory response in the respiratory mucosal membrane. IL-33 binds to its receptor ST2 on memory Th2 cells and induces epigenetic changes of the IL-5 gene, resulting in the generation of IL-5-producing Th2 cells (47). Thus, Th2 differentiation, which is characteristic of allergy, is triggered by phosphorylation of STAT6 signal transducers and expression of GATA3 and Th2 cytokines, including IL-4 (47).

Demethylation of the IL-4 promoter leads to allergic sensitization (48). Th1 differentiation is in turn triggered by phosphorylation of STAT4 signaling, and expression of the transcription factor T-bet and cytokine. For a more detailed description of epigenetics and T cell development, see Box 2. Asthmatic individuals show a lower histone deacetylase (HDAC): histone acetylase (HAT) ratio, i.e., a relative decrease of HDAC enzymes, which is corrected by proper anti-asthma treatment (50). The DNA methylation status of Foxp3 is regulated within a highly conserved region within the CpG-rich Treg-specific demethylated region with a differential Foxp3 demethylation status in children with an active cows milk allergy (CMA) and acquisition of immune tolerance (51).

Box 2 **Epigenetics and T-cell subset development.**

**Figure:**
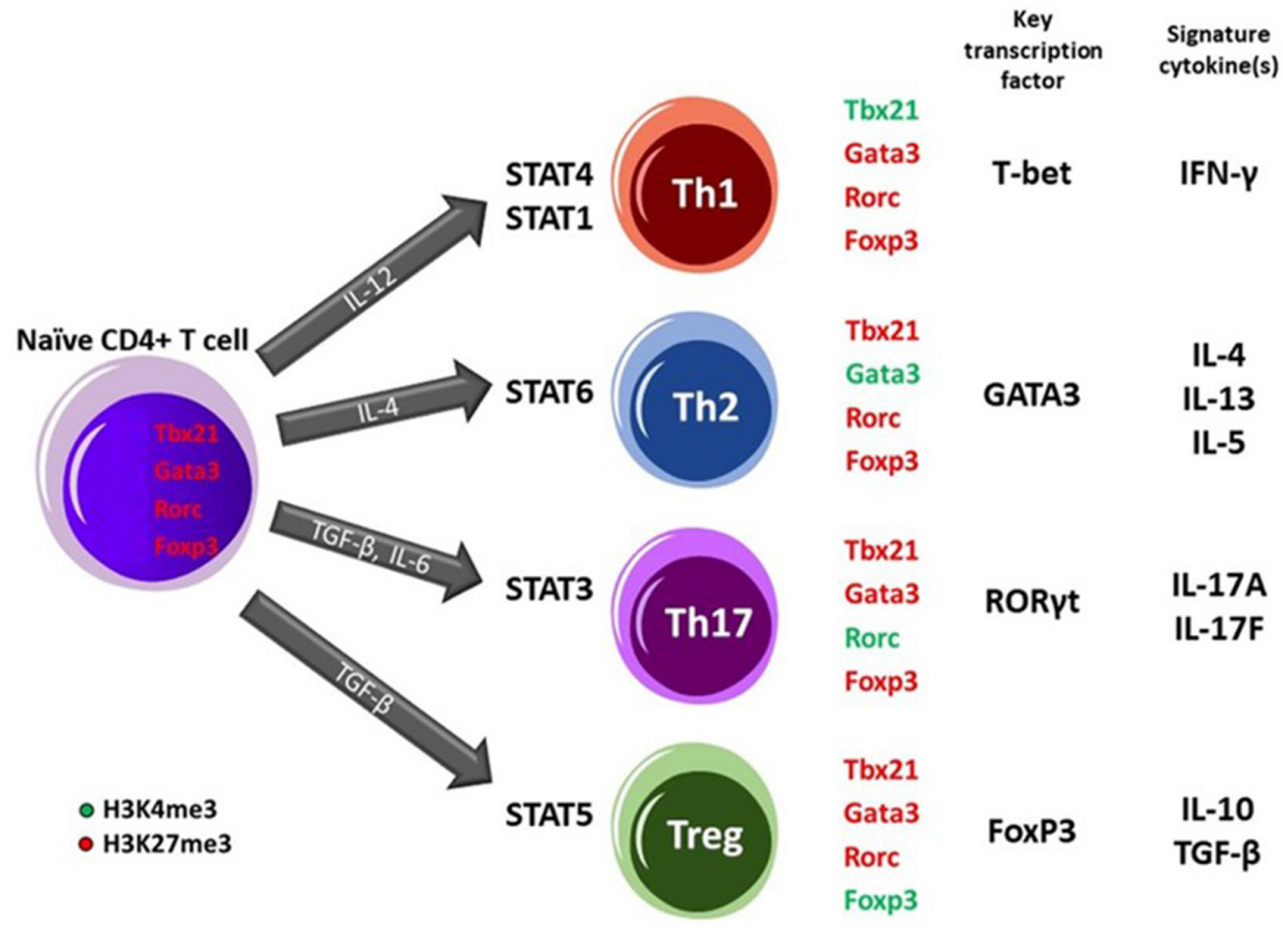

The differentiation of naïve CD4+ T cells upon antigen exposure into effector T helper (Th) subsets (Th1, Th2, and Th17) or induced regulatory T (iTreg) cells relies on epigenetic regulation and the establishment of cell-fate programs (40, 41). DNA methylation and chromatin modifications at pivotal loci in Th cells such as IFN-γ, IL-4 and, Foxp3 contribute to the formation of stable, heritable gene expression patterns. Methylation of CpG dinucleotides specially at promoter or other regulatory regions of genes is generally considered a repressive feature causing silenced genes what mostly seen in (embryonic) stem cells. Targeted loci DNA demethylation is required during early or late hematopoietic cell differentiation (41, 42). For instance, DNA demethylation plays a role in the expression of Th2 cell-related cytokine, IL-4 (43) and, Treg cell-related regulators (44, 45). Besides DNA methylation, histone modifications including acetylation and methylation have a role in the development of Th cell lineage. Histone acetylation, associated with the control of gene expression by condensing or relaxing the chromatin structure to repress or activate transcription, respectively, regulates the expression of several inflammatory mediators of the immune system. In this regard, modifications of histones occur in the enhancer and promoter regions of the STAT4 and STAT1 transcription factor binding sites upstream of the IFN-γ and TBX21 (T-bet) gene to direct Th1 differentiation. In contrast, activation of STAT6 in response to IL-4 occurs leading to the expression of IL-4 and GATA3 transcription factor genes in Th2 differentiating cells. Driving naïve CD4+ T cells toward Th17 phenotype requires STAT3 activation followed by expression of RORC gene encoding RORγt transcription factor and subsequently the production of IL-17 cytokines. Alternatively, upon naïve CD4+ T cells exposure to TGF-β, STAT5 transcription factor engages leading to changes in Foxp3 gene promoter site and commitment of cells into Treg fate. These specific histone modifications lead to engagement of lineage-specific key transcription factors which ensures Th phenotype stabilization and prevents the cells from skewing toward alternative commitments (35, 42, 46).

## Effects of Early Life Nutrition on Allergic Disease

The WHO recommends exclusive breastfeeding for infants during the first 6 months of life, and that it should be given alongside complementary feeding up until children are 2 years old (52). If mothers are unable to breastfeed, many children receive early life nutrition alternatives that are based on bovine milk. Therefore, this section of the study is focused on breast milk, bovine milk, and their components.

### Effects of Maternal Diet in Pregnancy and Breastfeeding on Allergic Disease

There is increasing evidence to suggest that the maternal diet during pregnancy and breastfeeding can have sustained effects on immunological outcomes in the infant and even have ramifications for their health later in life. The maternal diet can modify some immune supporting micronutrients in breast milk, such as the fat-soluble vitamins A and D, as well as the water-soluble B vitamins, and polyunsaturated fatty acids (PUFA), but maternal diet does not influence other components such as iron and zinc (53). Although there is some conflicting data, supplementation of maternal diet with vitamins and micronutrients during pregnancy and breastfeeding does not seem to prevent infections and allergies in offspring (54, 55).

#### Supplementation of Maternal Diet With PUFA

Long-chain PUFA (LCPUFA) induce inflammation by modulating inflammatory mediators like prostaglandins and immunomodulatory factors like IL-10 and TSLP (56). Consumption of omega-3 PUFA correlates with the inhibition of TLR4 signaling and thereby the production of inflammatory cytokines (IL-1, IL-6, and TNFα), which is reflected by a lower risk of allergies, whereas consumption of saturated fats and omega-6 PUFA, a potential trigger for TLR4-induced inflammation, has been associated with a higher risk of allergies. In addition, PUFA supplementation during pregnancy was associated with a reduction in allergic outcomes after birth (57, 58), but not when it was supplemented to infants (8, 59–61), suggesting that pregnancy is an important time that influences the development of the immune system.

#### Supplementation of Maternal Diet With Pre-/Probiotics

Probiotics are living microorganisms which, when administered in adequate amounts, confer a health benefit to the host. They generally exist of *Lactobacillus, Bifidobacterium*, or *Escherichia* species, which are commonly found in a normal microbiota. Prebiotics are mostly dietary fibers that are non-digestible food ingredients and beneficially affect the host's health by selectively stimulating the growth and/or activity of some genera of microorganisms in the colon, generally lactobacilli and bifidobacteria.

Intestinal microbiota strongly influence the maturation of the immune system (62) and particularly the development of immune tolerance, because they affect the Th1/Th2/Th17/Treg balance. The microbiota composition is modulated by dietary components that help shaping and timing of the composition of the early microbiome (63, 64). In addition, microbiota can be transmitted directly into the uterus during fetal development, passage through the birth canal or during cesarean-section, breastfeeding, and when providing care to the offspring (65, 66).

Food supplements, which are often termed functional foods, have been used to alter, modify, and reinstate pre-existing intestinal microbiota (67). Supplementation of prebiotics, probiotics, and synbiotics (68–74), as well as PUFA (58, 69, 75–77) during pregnancy and breastfeeding, may reduce eczema in infants. This is further supported by preclinical studies, which indicated that supplementing the maternal diet with specific pre- or probiotics affects milk composition (78) and that supplementing non-digestible oligosaccharides diminished allergic disease in offspring (79–81). This may, in part, be linked to the production of SCFA by the intestinal microbiota (82–86). Even though maternal diet during pregnancy and breastfeeding can modulate the prevalence of allergy in the offspring, the potential role of breastfeeding in allergy prevention is still under discussion, as it seems to be linked to variations in breast milk composition rather than to breastfeeding *per se* (53, 87).

### Effects of Consumption of Raw Milk and the Farming Environment

Most of our current knowledge on the effects of environment and diet on epigenetics and early life immune function is based on epidemiological findings, which indicate that children who grow up on farms have a decreased risk of developing allergies, especially asthma. Allergies are multifactorial, Th2-driven diseases that are triggered by gene-environment interactions. Environmental factors can interact with genes involved in asthma and allergy development via epigenetic mechanisms, such as DNA methylation and histone modifications. These epigenetic mechanisms can regulate gene expression by modifying the accessibility of the DNA to transcription enzymes without altering the DNA nucleotide sequence (30, 33). In addition to the consumption of raw cow's milk (22–29), contact with livestock and animal feed along with other farm-related exposures have shown independent protective effects, indicating that a farm/country lifestyle can contributes to a reduced risk of asthma and allergies in children (25, 27, 88–90). Interestingly, the timing of these exposures seems to be crucial, with the strongest effects observed for exposures that occurred *in utero* and during the first year of life (23, 91, 92). Since the protective “farm effect” was demonstrated to sustain into adult life (25), effects might be mediated via epigenetic inheritance/regulation.

Several epigenome wide-association studies concerning allergies have been performed and reviewed (30). These studies showed that allergic disease is accompanied by changing DNA methylation patterns in Th2, Th1, Th17, Th9, and Treg subsets in the affected tissues. DNA methylation changes by demethylation and increased FoxP3^+^ regulatory T cell numbers in peripheral blood mononuclear cells were shown in 4.5-year-old farm children (93). These regulatory T cell numbers were negatively associated with doctor-diagnosed asthma. It remains to be seen if these changes also precede the onset of allergic disease and can be predictive for allergy development, but questions remain as to how are these epigenetic changes induced. It has been suggested that the epigenome is affected by the farm environment. The first indication for a potential role of epigenetic regulation in the protective “farm effect” was provided by Slaats et al. who demonstrated that DNA methylation of the promoter region of CD14 in placentas of mothers living on farms was lower compared to mothers not living on a farm (94). These lower DNA methylation levels were reflected in higher CD14 mRNA expression levels (95). Interestingly, a higher expression of the CD14 gene was also observed in farmers' children (96). Prenatal farm exposure was also associated with increased gene expression of other innate immune receptors, such as TLR5, TLR7, TLR8, and TLR9, at birth (97, 98) and TLR2 and TLR4 in farm-raised children at school age (95, 96). Maternal exposure to farm environments increases the number of T regulatory (Treg) cells in the cord blood of infants, which is associated with decreased Th2 cytokines and may be linked to demethylation at the FOXp3 promoter (99). Whether epigenetic inheritance is underlying these effects requires further investigation. Further evidence that the farm environment affects the epigenome was provided by a pilot study which showed hypermethylation of genes related to IgE regulation and Th2 differentiation in cord blood from farmers' as compared to non-farmers' children (100). Interestingly, at least part of the protective effect triggered by those factors has been ascribed to the farm bacteria, for instance, *Acinetobacter lwoffii* (101, 102), with a pivotal contribution of downstream epigenetic mechanisms, specifically histone modifications (103).

### Milk Components

Human milk contains a unique combination of lipids, proteins, carbohydrates, vitamins, and minerals and thereby provides an ideal source of nutrition for the healthy growth and development of a newborn (104). However, human milk is more than nutrition as it also contains bioactive components that can modulate the immune system, such as immunoglobulins, lactoferrin, human milk oligosaccharides (HMO), long-chain fatty acids, and anti-inflammatory cytokines (18, 105, 106). Most of the immunologically relevant components in breast milk are also found in bovine milk (18). Several key components of breast milk that are not present at high enough levels in bovine milk are added to infant formula to provide the crucial nutrients needed. These include prebiotics or even single HMO like 2'-fucosyllactose (as an alternative to the complex mixture of HMO in breast milk), lactoferrin, PUFA, vitamins, and minerals.

#### Non-digestible Milk Oligosaccharides

One of the major differences between human breast milk and bovine milk is the amount and diversity of the HMO, i.e., complex, non-digestible oligosaccharides (107, 108). The HMO in breast milk constitutes about 20% of the milk saccharides next to the major carbohydrate in milk, lactose. Human breast milk contains ~5–15 mg/ml of these non-digestible HMO, consisting of up to 200 or more unique structures. In contrast, bovine milk only contains a few of these oligosaccharides, at much lower levels. One injected, HMO survive passage and digestion through the stomach and small intestine and reach the colon, where they are fermented into SCFA like acetate, butyrate, and propionate (107, 108). In addition, they shape the microbiota by selectively enhancing the growth of bifidobacteria and lactobacilli. These SCFAs serve as an energy source for colonic intestinal tissue and shape the interactions between the host and its gut microbiota. Furthermore, SCFA reduces intestinal pH, limit outgrowth of *Enterobacteriaceae*, and support intestinal barrier function. HMO is the key factor in shaping the development of immunity and early microbiota after birth. HMO have effects on microbiota and infections (107, 108). Of these, 2'-fucosyllactose is the HMO that is most abundantly present in breast milk and has therefore been chosen as the first HMO that was introduced in infant nutrition in 2018.

Prebiotics are non-digestible oligosaccharides like galacto-oligosaccharides (GOS) and fructo-oligosaccharides (FOS), and have widely been used in infant nutrition to mimic the bifidogenic- and SCFA-inducing effect of HMO. There is some evidence that prebiotic oligosaccharides in infant nutrition may prevent eczema in infants (109–112). It is not clear if these effects also extend to the prevention of other allergic diseases, as only one study to date has reported the effects of prebiotics on asthma and food allergy (113). For probiotics, effects are also seen when they are added in infant nutrition (68). As can be seen in detail in Lomax and Calder (114), several studies have reported that infant formula supplemented with prebiotics have a trend toward or even a significant preventive effect on the occurrence of gastrointestinal infections. Trends toward decreased fever episodes, antibiotic use, and upper respiratory tract infections (URTI) have been described. Two studies, by Bruzzese et al. and Arslanoglu et al. and performed with scGOS/lcFOS, supplemented very young infants from early after birth for 6–12 months (115, 116). Both studies showed a significant reduction in gastroenteritis (115) and a reduction in the total number of infections (116). A study from Westerbeek et al., in which scGOS/lcFOS were combined with acidic oligosaccharides (pAOS) showed a non-significant tendency toward fewer serious infections (117). This study was, however, conducted over a shorter time period, and the infants were preterm. In two other studies infants were older than 6 months (118, 119) were supplemented with oligofructose, one did not show an effect on diarrhea, whilst the latter observed a protective effect against diarrhea. Since these components and their effects have been reviewed in detail previously, we will not address them in detail here, and will instead, only focus on their potential epigenetic and long-lasting immune health effects.

#### Bioactive Components Besides Non-digestible Oligosaccharides

Both human milk and bovine milk contain many other bioactive components that can modulate immune function [reviewed in (18, 19, 105–107)]. The components in human and in bovine milk that can be isolated in large quantities have largely been studied as separate entities, because they are potential infant nutrition ingredients. Several of these components, such as transforming growth factor-β (TGF-β) (120), bovine lactoferrin (121–124), bovine alkaline phosphatase (19, 125), bovine osteopontin (126, 127), and the milk fat globular membrane (MFGM) (128), as well as milk exosomes (39), have been linked to immunological outcomes with varying levels of evidence (infection, allergy). Another milk component that may have more sustained immunological effects are bovine IgG antibodies. Where IgA is the predominant immunoglobulin isotype in breast milk, bovine milk has a larger amount of IgG (129). Bovine milk IgG (bIgG) has been shown to bind to aeroallergens (130) as well as to respiratory pathogens such as respiratory syncytial virus (RSV), and can inhibit infection of human cells with human RSV (131). Through the formation of immune complexes, bIgG can enhance RSV-specific T cell responses (132). Similarly, bovine colostrum, which is a rich source of IgG can prevent the infection of mice with RSV (133). Different from adaptive immunity, innate immunity was until recently believed to lead to immune memory. However, vaccination studies have shown that after vaccination—that is associated with cross-protection to other pathogens—the innate immune response is increased to the vaccine, but also other pathogens (134, 135). The mechanism of this was elucidated in several mechanistic studies and was shown to be dependent on epigenetic modification of monocytes and macrophages (136–139). Even though epigenetic modification was not directly shown, bovine IgG can induce trained immunity in monocytes (140). In addition to possibly preventing some of the epigenetic modifications induced by infection with respiratory viruses, which would be the result of the lower prevalence of respiratory tract infections (21), bovine IgG may also directly modify subsequent innate immune responses in infants.

## (Epigenetic) Effects of Human Breast Milk and Bovine Milk on Allergy Outcomes Later in Life

Several epigenome wide-association studies on allergies have been performed, as reviewed elsewhere (30). These studies have shown that allergic disease is accompanied by changing DNA methylation patterns in Th2, Th1, Th17, Th9, and Treg subsets in affected tissues. The epigenetic mechanism behind T cell subset differentiation is strongly affected by essential micronutrients (folate, vitamins B2, B6, and B12, methionine choline, and betaine) (141), bioactive food components (tea polyphenols, genistein from soybean, isothiocyanates from plant foods, curcumin, and curcumin-derived synthetic analogs) (142), total diet (fiber, protein, fat, and hormones) (143), ethanol, and carbohydrates (144). Dietary compounds, especially vitamin D, folate, and zinc, also have the potency to interfere with DNA methylation and thereby steer the Th1-Th2 balance. In addition to these effects on DNA methylation, prenatal supplementation with PUFA or maternal levels of folate, and microbiota-derived SCFA have been associated with changes in histone acetylation patterns at important T cell differentiation regulating genes (Box 2). After birth, these immunomodulatory dietary components are also transferred to the newborn via breast milk.

### Epigenetic Effects of Breastfeeding, Raw Milk, and Exposure to the Farming Environment in Early Life

As already mentioned, the mechanisms underlying the anti-allergic effects of human milk are most probably complex, as human milk contains not only nutritional substances but also functional molecules including polysaccharides, cytokines, proteins, and other components forming a real biological system which can modulate and shape the innate and adaptive immune responses of the infant in very early life (104, 145). If and how those components affect the epigenetic status of the growing child and what consequences this has for allergy development need to be addressed in future studies. Considering the observations made about farm milk (see below), as well as indications that breastfeeding may be capable of changing DNA methylation patterns in the offspring (146), such studies are justified.

Epigenetic modulation of the Foxp3 gene by farm milk was demonstrated in an animal model. In this study, exposure to raw, unprocessed, cow's milk for 8 days, increased histone acetylation of Foxp3 in splenocyte-derived CD4+ T cells compared to processed milk exposure (147). In the same study, mice were subjected to an ovalbumin-induced food allergy model after milk exposure and, interestingly, histone acetylation of Th2 genes was lower in raw milk-pretreated mice compared to processed milk-pretreated mice. These mice also showed a reduction in food allergic symptoms (147). As for farm exposure, exposure to raw milk in the first year of life was also associated with changes in gene expression of the innate immune receptors (98). Moreover, it was demonstrated that a polymorphism in the CD14 gene influenced the protective effect of raw cow milk consumption on allergic diseases (148). DNA demethylation and increased Foxp3+ in the regulatory T cell numbers in the peripheral blood mononuclear cells of 4.5 year-old children were also shown in farm children (93). These regulatory T cell numbers were negatively associated with doctor-diagnosed asthma. It remains to be seen if these changes also precede the onset of allergic disease and can be predictive of allergy development.

There is evidence that the epigenome is affected by the farming environment. The first indication for a potential role of epigenetic regulation in the protective “farm effect” was provided by Slaats et al. who demonstrated that DNA methylation of the promoter region of CD14 in placentas of mothers living on a farm was lower compared to mothers not living on a farm (94). These lower DNA methylation levels were reflected in higher CD14 mRNA expression levels (95). Interestingly, a higher expression of the CD14 gene was also observed in the children of farmers (96). Prenatal farm exposure was also associated with increased gene expression of other innate immune receptors, such as TLR5, TLR7, TLR8, and TLR9, at birth (97, 98) and TLR2 and TLR4 in farm-raised children at school age (91, 96). Maternal exposure to farming environments increased the number of Treg cells in the cord blood of infants, which is associated with decreased Th2 cytokines and may be linked to demethylation at the Foxp3 promoter (50). Whether epigenetic inheritance is the underlying cause of these effects requires further research. Additional evidence that the farm environment affects the epigenome was provided by a pilot study that showed DNA hypermethylation of genes related to IgE regulation and Th2 differentiation in cord blood from the children of farmers as compared to the children of non-farmers (100).

### Epigenetic Effects of miRNA Containing Extracellular Vesicles (Exosomes)

Interestingly, both human and cow's milk contain extracellular vesicles, or exosomes, that are resistant to the acidic environment in the stomach and RNAses in the GI tract. These exosomes contain a variety of especially immune function-related microRNAs (miRNAs). miRNAs represent short noncoding RNA molecules that control 40–60% of the total gene expression by inducing mRNA degradation and/or post-transcriptional inhibition of translation. As a result, specific miRNA can silence selective gene expression. The expression of a single gene can be regulated by several miRNAs, and likewise, a single miRNA can regulate over 100 genes (32, 149). This activity thereby constitutes an epigenetic mechanism by which nutritional factors can influence immune activity or the induction of tolerance by affecting the Th1-Th2 balance. Bovine milk exosomes are taken up by human macrophages (150) and epithelial cells (151, 152), exosomes become systemically available in the body of laboratory animals upon oral delivery (153), and bovine miRNA are detectible in the blood after drinking pasteurized milk (154). However, systemic availability could not be demonstrated for breast milk derived exosomes (155) or vegetable derived miRNA (156). Breast milk-derived exosomes were described in 2007 to enhance Treg development *in vitro* (157). Based on miRNA content, bovine milk exosomes contain immunoregulatory miRNAs, like miRNA155, that are involved in the development of Tregs and are thought to play a role in the effect of raw milk consumption on asthma (39). In addition to allergy, orally delivered bovine milk exosomes ameliorated arthritis in a murine model (158), and recent evidence also links milk exosomes to the prevention of necrotizing enterocolitis and intestinal damage in *in vitro* and *in vivo* investigations (159, 160). These studies suggest that miRNAs in human and raw bovine milk exosomes may have epigenetic effects in infants.

### Epigenetic Effects of SCFA

Several studies have implicated the SCFA butyrate, propionate, and acetate as epigenetic modifiers of early life immunity, especially in the development of asthma (161). In addition to regulating Treg differentiation and histone acetylation, SCFAs can induce effector T cell differentiation in secondary lymphoid organs by inhibiting endogenous HDAC activity independent of activation of G-protein-coupled receptor (GPCR). In more detail, SCFA can modulate diverse cell processes by two mechanisms, either via interacting with the GPCR (GPR43, GPR41, GPR109A) on the plasma membrane or following a receptor-independent entrance to the cells (162). SCFA entry occurs through passive diffusion or actively by the involvement of two transporters, namely, monocarboxylate transporter 1 (MCT1/SLa16a1) and sodium-coupled monocarboxylate transporter 1 (SMCT1/SLc5a8). These receptors and transporter molecules are widely present in immune and non-immune cells (162, 163). This effect is highly pronounced for butyrate and to a lesser extent for propionate and acetate (164–166). HDAC inhibition allows HATs activity leading to histone hyperacetylation and subsequently an altered gene expression (37) which might, for instance, result in the proliferation of Treg cells (167–169). The significance of this mechanism is illustrated by the fact that bovine, but not human, milk triglycerides contain a relatively high concentration of the SCFA butyrate (18). Altogether, present evidence implies that HDAC inhibitory activity of SCFA might be cell and tissue dependent, and the gene expression pattern is related to the cellular stage and other environmental signals. If bovine milk consumption is associated with decreased allergy prevalence, does this also mean that milk components can affect epigenetic mechanisms? There is no *in vivo* evidence that the induction of SCFA by sialyllactose when ingested in bovine milk, but sialyllactose has been reported to induce SCFA production in *in vitro* fecal microbiota cultures (170) and may thus affect histone acetylation in infants. A high fiber diet (resulting in SCFA production in the colon) or direct feeding of SCFA has been shown to prevent airway inflammation in animal models (84, 85), and SCFA levels in fecal samples of children associated inversely with sensitization to aeroallergens (171, 172).

In addition to allergies, intestinal immunity can also be influenced by microbiota-derived metabolites. For example, tryptophan metabolites can act as aryl hydrocarbon receptor (AhR) ligands, inducing IL-22 and antibacterial peptide production (173), SCFA can directly support the intestinal epithelial barrier, and bile acids can also be metabolized by the microbiota and influence intestinal barrier function and immunity (174). Two studies reported a decreased risk of wheezing in infants because of high maternal dairy intake (175, 176). Taken together, alterations in the local cellular microenvironment and the microbiome (56) allow milk to induce epigenetic changes in both maternal and neonatal nutrition-mediated genes, which can ultimately affect immune programming in the offspring (177).

## Conclusions

This review summarizes current knowledge on the potential effects of human and bovine milk on neonatal immunity and epigenetic programming and its possible consequences on the development of allergies in early childhood and beyond (see Figure 1).

Breast milk is the food of choice for newborns and infants. When breast milk is not sufficiently available, cow's milk based formula is the best alternative, and thus cow's milk has become an integral part of early life diet.

Several epidemiological studies that have shown that exposure to a farm environment as well as to raw/unprocessed cow's milk in the prenatal period and early childhood is associated with protection against the development of asthma and other allergies later in life. Many cow's milk components have been shown to have similar effects on human immune cells as their breast milk counterparts.

Some of the molecular pathways that may explain the association between the consumption of raw milk asthma and allergy may be linked to epigenetics. Epigenetic mechanisms like DNA methylation, but also histone modifications, and non-classical epigenetics represented by miRNA may all contribute to the effects induced by raw cow's milk.

However, milk and dairy products are subject to industrial processing to ensure microbiological safety. As a result, milk proteins can be denatured, and lose their functional activity. In addition, glycation of milk proteins is thought to increase the risk of developing cow's milk allergy, illustrating that preserving milk proteins and preventing glycation may be important innovations to help prevent allergies.

Based on what is currently known on immunological and epigenetic effects that can be exerted by human and different types of bovine milk, future research should focus on enhancing the functional (immunological as well as epigenetic) activity of milk components in early life nutrition, and on establishing epigenetic markers of immunological responses to milk. These could be especially important for diagnostic purposes and assessing the risk of developing CMA. Knowledge gathered during studies on the epigenetic effects of milk can be used in the future to drive the development of preventive or therapeutic anti-allergic strategies based on components that affect epigenetic mechanisms.

Finally, the continuation of epidemiologic and mechanistic studies on the effects of the components of breast and bovine milk on human immune function and health will increase our knowledge and help in finding potential applications that may help prevent allergies in the neonatal period.

## Author Contributions

All authors contributed to the writing of the manuscript.

## Conflict of Interest

BE and JG are partly employed by Nutricia Research. RN is employed by FrieslandCampina. The remaining authors declare that the research was conducted in the absence of any commercial or financial relationships that could be construed as a potential conflict of interest.
